# Fall Risk and Psychotropic and General Medication Use in Dementia with Neuropsychiatric Symptoms

**DOI:** 10.1002/alz70857_102061

**Published:** 2025-12-24

**Authors:** Sena Gok, Salma Amarin, Nicole Schoer, Amer M. Burhan, Sarah Colman, Li Chu, Simon Davies, Peter Derkach, Sarah Elmi, Philip Gerretsen, Ariel Graff‐Guerrero, Maria Hussain, Donna Kim, Zahinoor Ismail, Linda Krisman, Benoit H. Mulsant, Bruce G. Pollock, Soham Rej, Aviva Rostas, Lisa Van Bussel, Tarek K. Rajji, Sanjeev Kumar

**Affiliations:** ^1^ CAMH, Toronto, ON, Canada; ^2^ Centre for Addiction and Mental Health, Toronto, ON, Canada; ^3^ University of Toronto, Toronto, ON, Canada; ^4^ Ontario Shores Centre for Mental Health Sciences, Whitby, ON, Canada; ^5^ Ukrainian Canadian Care Centre, Toronto, ON, Canada; ^6^ West Park Health Care Centre, Toronto, ON, Canada; ^7^ Toronto Dementia Research Alliance, Toronto, ON, Canada; ^8^ Department of Psychiatry, Temerty Faculty of Medicine, University of Toronto, Toronto, ON, Canada; ^9^ Campbell Family Mental Health Research Institute, Centre for Addiction and Mental Health, Toronto, ON, Canada; ^10^ Queen's University, Kingston, ON, Canada; ^11^ University of Calgary, Calgary, AB, Canada; ^12^ Hotchkiss Brain Institute, University of Calgary, Calgary, AB, Canada; ^13^ Hotchkiss Brain Institute, Calgary, AB, Canada; ^14^ Western University, London, ON, Canada; ^15^ McGill University, Montreal, QC, Canada; ^16^ Adult Neurodevelopment and Geriatric Psychiatry Division, CAMH, Toronto, ON, Canada; ^17^ UT Southwestern Medical Center, Dallas, TX, USA; ^18^ Temerty Faculty of Medicine, Division of Psychiatry, University of Toronto, Toronto, ON, Canada; ^19^ Toronto Dementia Research Alliance, University of Toronto, Toronto, ON, Canada; ^20^ Temerty Centre for Therapeutic Brain Intervention CAMH, Toronto, ON, Canada; ^21^ Temerty Faculty of Medicine, University of Toronto, Toronto, ON, Canada

## Abstract

**Background:**

Polypharmacy is common in older individuals with Alzheimer's disease and related dementia (ADRD) who present with neuropsychiatric symptoms (NPS). While it is associated with increased fall risk, specific impact of psychotropic and general medication use on fall risk in this population is not clear. We evaluated fall risk associated with psychotropic and general medication use in patients with ADRD and NPS while controlling for other clinical factors.

**Method:**

We used the following data from participants with a clinical diagnosis of AD and clinically significant NPS in the Standardizing Care for Neuropsychiatric Symptoms and Quality of Life in Dementia (StaN) study (ClinicalTrials.gov/NCT03672201): number of psychotropic and general medications and scores on the Cohen Mansfield Agitation Inventory (CMAI) scores, Cumulative Illness Rating Scale‐Geriatric (CIRSG), Functional Assessment Staging Tool (FAST). and Morse Fall Scale (MFS). Linear regression analyses evaluated the associations between psychotropic or general medication use and fall risk (MFS) while adjusting for age, CIRS‐G, CMAI, and FAST scores.

**Result:**

185 participants (female: 96 (52.5%)) were included, with mean (SD) age of 80.5 (9.8) years, mean FAST score of 9.1 (SD: 0.65; median stage: 6e). Psychotropic or general medications and CMAI or CIRS‐G scores were not significantly associated with MFS, but FAST stage of dementia was.

**Conclusion:**

Fall risk in AD may be influenced more by functional and cognitive impairment than by medication use. These findings should be considered in the context of unique characteristics of this cohort that include a severe stage of dementia and the presence of significant NPS, and may inform fall risk management strategies in this population.

**References**

Gallagher, E., Mehmood, M., Lavan, A., Kenny, R. A., & Briggs, R. (2023). Psychotropic medication use and future unexplained and injurious falls and fracture amongst community‐dwelling older people: Data from TILDA. *European Geriatric Medicine, 14*(4), 455–463.

Zarei S, Choudhury S, Burhan AM, Chu L, Colman S, Derkach P, et al. Determinants of polypharmacy in patients with behavioral and psychological symptoms of dementia. Alzheimers Dement. 2021;17(S6).

